# The Role of Prior Achievement as an Antecedent to Student Homework Engagement

**DOI:** 10.3389/fpsyg.2019.00140

**Published:** 2019-02-01

**Authors:** Isabel Piñeiro, Iris Estévez, Carlos Freire, Ana de Caso, Alba Souto, Mercedes González-Sanmamed

**Affiliations:** ^1^Department of Psychology, University of A Coruña, A Coruña, Spain; ^2^Department of Pedagogy and Didactics, University of A Coruña, A Coruña, Spain; ^3^Department of Psychology, Sociology and Philosophy, University of León, León, Spain

**Keywords:** homework, academic achievement, motivational engagement, cognitive engagement, behavioral engagement, primary education

## Abstract

The benefits of homework on student learning and academic achievement, to a large extent, depend on the degree of student engagement. Motivational engagement (my intention or why I do the homework), cognitive engagement (how I get involved in homework), and behavioral engagement (how much homework I do, how much time I devote to it, how I manage that time) are key aspects that condition the quality of the process of doing homework, learning, and academic achievement. Prior academic achievement is one of the variables that seems to be positively related to student engagement (both due its motivational component and to the training to do homework). The main purpose of this work was to study in detail this relationship in students of the last stage of Primary Education (*N* = 516). The results showed that (i) as achievement levels rise, the use of a shallow focus to doing homework decreases (and the use of a deep approach increases); (ii) there is also a progressive increase in the amount of homework done and in the management of the time dedicated to homework. On another hand, as in previous research, (iii) no relationship was observed between the levels of prior achievement and the amount of time spent doing homework.

## Introduction

In recent years, extensive research aimed at analyzing the predictive value of diverse variables related to academic achievement has been developed. Although many of these predictive variables are of a cognitive and motivational nature (e.g., Dettmers et al., 2010; Miñano et al., 2012), there is no doubt that prior achievement and, especially, students’ experiences of success and failure, are the main predictor of their future academic career.

Indeed, students’ prior achievement has been widely viewed as a strong predictor of their academic success (Kitsantas and Zimmerman, 2009; Schneider and Preckel, 2017). Likewise, previous successful experiences can lead to important benefits in motivational, behavioral, and affective areas (Regueiro et al., 2017). Thus, in a study conducted by Goetz et al. (2008), it was found that prior achievement in the subject of mathematics positively predicted enjoyment of and positive feelings toward this subject. In this sense, Pan et al. (2013) observed that students with the highest levels of academic achievement were the most intrinsically motivated to do homework. In other studies (e.g., Goldberg and Cornell, 1998; Garon-Carrier et al., 2016) prior achievement was associated with later intrinsic motivation over time, whereas the findings of other works (e.g., Taylor et al., 2014) provide some evidence that the relations between academic motivation types and achievement may be reciprocal.

In addition, motivational variables largely determine students’ homework engagement (Valle et al., 2015b). In fact, during the process of doing homework, students must adapt to the demands of its performance, which requires not only planning and setting priorities, but also managing time, and coping with distractions, as well as controlling motivation and emotion.

In this way, the relevance of this study is to determine the role of prior academic achievement and, consequently, the successful experiences derived thereof, in the degree of motivational and cognitive engagement (operationalized in the student’s approach to doing homework) and also students’ behavioral engagement to homework (amount of homework done, amount of time spent on homework, and time optimization). We draw on the idea that motivation is a necessary but insufficient condition to activate cognitive and behavioral engagement toward a task (Appleton et al., 2006).

### Motivational and Cognitive Engagement to Homework: Approach to Homework

The process of doing homework refers to what students do when faced with homework; that is, how they do their homework and how they manage personal and environmental resources when they do it. Therefore, rather than focusing on the amount of homework that should be assigned or on the time that should be spent on homework, the focus would be on the process, that is, on the quality of students’ performance when faced with homework (Dettmers et al., 2010). In fact, in students’ process of doing homework, *how* should matter more than *how much* (Fernández-Alonso et al., 2016; Rosário et al., 2018).

Quality in the process of homework preparation is considered in this paper as the higher or lower degree of depth with which the student deals with the tasks. Everything seems to indicate that the approach to homework used (motivational and cognitive engagement) not only influences the final performance of homework but also the quality of the process of doing homework. The approach to homework adopted by the student is one of the aspects that can provide more information about the motivation for doing homework and also the strategies and resources (consistent with those motivations) implemented for this purpose (Valle et al., 2017).

Students who adopt a deep approach will engage in homework with the intention of learning and reinforcing the contents acquired in class, trying to resolve the doubts that arise while doing homework, and relating the homework to what they learned previously (Valle et al., 2015b). Conversely, those who adopt a shallow approach will do homework because they feel obligated, and their priority will be to finish it as soon as possible in order to devote themselves to other more amusing activities. They will only be concerned about doing homework because they must hand it in and correct it in class, but not because they think that its performance contributes to consolidating or improving their learning (Valle et al., 2017).

Most of the studies have shown that the deeper the students’ approach to learning, the better the quality of their learning outcomes (Cano et al., 2014). In addition, whereas the shallow approach is related to poor academic results (Rosário et al., 2010), the use of a deep approach is associated with high levels of understanding and achievement (Biggs, 1993), an intrinsic interest in learning, and high levels of comprehension (Trigwell et al., 2005). In the same vein, Bembenutty and White (2013) found that, when students do homework with a deep approach, showing interest in the task and a positive attitude, they tend to have good academic achievement in the different subjects.

### Behavioral Homework Engagement: Amount of Homework Performed, Amount of Time Spent on Homework, Homework Time Management

Behavioral homework engagement manifests in the students’ degree of engagement and active participation in the process of preparation of homework. A part of the recent research (e.g., see Rosário et al., 2009; Núñez et al., 2015a,b; Valle et al., 2015a) includes the study of three variables related to behavioral engagement: the amount of homework done, the amount of time spent on homework, and the optimization or management of that time.

The amount of teacher-assigned homework done is often positively related to improved academic achievement (Valle et al., 2016). In fact, some studies have found that students who do their homework obtain better academic grades than those who do not do it (Cooper, 1989; Trautwein et al., 2002).

As with the amount of homework, when referring to the time spent on homework, research advises differentiating between the amount of time spent on homework and the management or optimization of that time. Therefore, the adequate management of the time and effort invested is much more important for homework than the amount of time spent (e.g., number of hours dedicated to homework). In fact, in a study by Trautwein (2007), it was found that the relations between the amount of time spent on homework and academic achievement were moderate at the group level but negative at the individual level. These results may indicate that spending too much time doing homework can reflect insufficient prior knowledge or difficulty to understand the contents addressed in the homework. In other similar studies (e.g., see Fernández-Alonso et al., 2015), it was considered that many students who spend more time on homework probably have major gaps in their learning and concentration problems.

Therefore, the amount of time spent on homework is a merely quantitative aspect of the hours that students spend doing homework, but in no case is this necessarily a reflection of the effort and quality of their dedication (Flunger et al., 2015). Hence, managing the time spent on homework is a challenge for students, as adequate time management has a positive influence on students’ academic success (Claessens et al., 2007), the completion of homework (Xu, 2005, 2011), and academic achievement (Eilam, 2001; Trautwein et al., 2015). In addition, students who manage their homework time well (but do not necessarily spend more time) are the ones with a deeper approach to homework (Valle et al., 2015b, 2016).

### Purpose of This Study

The main purpose of this work is to analyze how prior academic achievement conditions students’ motivational and cognitive engagement (the approach to homework) and behavioral homework engagement (amount of homework done, amount of time spent, and time optimization). It attempts to provide evidence showing that prior experiences of success and failure largely condition students’ academic achievement, which is also manifest in an improvement of the quality of motivational, cognitive, and behavioral homework engagement.

The study of this relationship can provide clear evidence about how prior academic achievement activates motivational (reasons for doing homework), cognitive (homework engagement), and behavioral factors (amount of homework done, amount of time spent, management of that time), which will ultimately determine the quality of the process of doing homework. The working hypothesis is that higher levels of prior achievement would be positively and significantly associated with motivational and cognitive engagement, characterized by a deep approach to homework aimed at understanding and meaning. In addition, we also expected that higher levels of prior achievement would be positively and significantly related to a greater amount of homework done and to better time optimization. On another hand, it was also hypothesized that high levels of achievement would be associated with low motivational and cognitive engagement, which defines the shallow approach to homework. In addition, it was expected that prior academic achievement would not be related to the amount of time that students dedicate to homework.

## Materials and Methods

### Participants

The sample, selected through intentional sampling, is made up of 516 students from four public schools of Primary Education of the Autonomous Community of Galicia (Spain). Two schools are located in urban areas, and the other two are, respectively in rural and semi-urban areas. Concerning gender, 49% (*n* = 253) are boys and 51% (*n* = 263) are girls. Their ages ranged between 9 and 13 years (*M* = 10.35; *SD* = 0.99), 38.2% (*n* = 197) were enrolled in 4th grade of Primary Education, 36.4% (*n* = 188) were in 5th grade of Primary Education, and 25.4% (*n* = 131) were in 6th grade of Primary Education.

### Instruments

#### Behavioral Homework Engagement

To measure behavioral engagement (the time dedicated to doing homework and the amount of homework done), we used the *Encuesta sobre los Deberes Escolares (EDE, Survey on School Homework)*, which has been used in recent studies (e.g., see Rosário et al., 2009; Núñez et al., 2015a,b; Valle et al., 2015a) to obtain this kind of data.

To measure the *daily time devoted to doing homework*, students responded to three items (α = 74) (in general, during a typical week, on a typical weekend), from the general sentence “How much time do you usually spend on homework?”, with the following response options: 1 = *less than 30 min*, 2 = *30 min to one hour*, 3 = *one hour to an hour and a half*, 4 = *one hour and a half to two hours*, 5 = *more than two hours*.

With regard to *optimizing the time spent on homework* (α = 0.79), this was measured through the responses to three items (in general, during a typical week, on a typical weekend) in which they were asked to indicate the level of optimization of the time normally spent on homework, using the following scale: 1 = *I waste it completely* (I am constantly distracted by anything), 2 = *I waste it more than I should*, 3 = *regular*, 4 = *I optimize it pretty much*, 5 = *I optimize it completely* (*I concentrate and I don’t think about anything else until I finish*).

Finally, estimation of the *amount of homework done* by students was obtained through responses to an item about the amount of homework usually done, using a 5-point Likert-type scale (1 = *none*, 2 = *some*, 3 = *one half*, 4 = *almost all*, 5 = *all*).

#### Motivational and Cognitive Homework Engagement

To measure motivational and cognitive engagement (approach to homework), we used an adaptation of the *Students’ Approaches to Learning Inventory* (Rosário et al., 2010, 2013), taking into account both the students’ age and the homework context. The questionnaire is composed of twelve items, of which six evaluated students’ motives and reasons for doing homework (three of them evaluate deep motives and another three shallow motives) and the other six items evaluate the cognitive strategies students implement when doing homework (three of them evaluate deep strategies and the other three shallow strategies). This instrument is based on existing research in the field of approaches to learning and study (e.g., Biggs et al., 2001), and provides information on two modes, or approaches, to homework: the shallow focus (α = 0.65) (e.g., item: “I usually do the homework, but rarely I notice how I’m doing”) and deep approach (α = 0.80) (e.g., item: “Before I start doing homework, I think about whether what was taught in class is clear and, if it is not, I review the lesson before starting”). Participants responded to the items on a 5-point Likert -type scale ranging from 1 (*totally false*) to 5 (*absolutely true*).

#### Prior Academic Achievement

Prior academic achievement was assessed through students’ report final card grades in Spanish Language, Galician Language, English Language, Knowledge of the Environment, and Mathematics. Average achievement was calculated with the mean grades in these five areas.

### Procedure

The study protocol was approved by the Research and Teaching Ethics Committee of the University of A Coruña. Data about the target variable were collected during school hours by personnel external to the center itself, after obtaining written informed consent of the management team, the students’ teachers, and the students’ parents, in accordance with the ethical standards established in the Declaration of Helsinki. Before applying the questionnaires, at a single time-point, participants were informed about the importance of responding sincerely to the different questions, emphasizing their completely confidential nature.

### Data Analysis

In order to comply with the objectives of the work, we performed a Multivariate Analysis of Covariance (MANCOVA), taking as the factor students’ prior academic achievement (with three levels: low, medium, and high) and as dependent variables those referring to the motivational and cognitive engagement (approaches do homework). The following criteria were used to determine the three levels of prior achievement: low achievement, up to the 33rd percentile; average achievement, from percentile 33 to 66; high achievement, as of the 66th percentile). Subsequently, we conducted another MANCOVA, taking prior academic achievement as the factor and, as dependent variables, those referring to behavioral engagement (quantity of homework done, amount of time spent, and optimization of that time). In order to statistically control for their possible effects, in both analyses, gender and grade were included as covariates.

As a measure of the effect size, we used the partial eta-squared coefficient ($\eta_{p}^{2}$), one of the most commonly used within educational research (e.g., Sun et al., 2010). The criterion established in the classical work of Cohen (1988) was used to interpret the effect sizes: null effect: $\eta_{p}^{2}$ < 0.01 (*d* < 0.09); small effect: $\eta_{p}^{2}$ = 0.01 to $\eta_{p}^{2}$ = 0.058 (*d* = 0.10 – *d* = 0.49); medium effect: $\eta_{p}^{2}$= 0.059 to $\eta_{p}^{2}$= 0.137 (*d* = 0.50 – *d* = 0.79); and large effect: $\eta_{p}^{2}$ ≥ 0.138 (*d* ≥ 0.80).

## Results

### Descriptive and Correlational Analysis

The relations between the variables and the descriptive statistics are shown in Table 1. Prior academic achievement was positively and significantly related to the deep approach, the amount of homework done, and management of the time spent on homework. However, academic achievement had a negative and significant relationship with the shallow focus and it had no relationship with the amount of time spent on homework. The deep approach showed a positive and significant relationship with the amount of homework done and with time management, but it was negatively and significantly related to the shallow focus, and had no relation with the time spent on homework. The shallow approach did not have any relationship with the time spent on homework, but it did present a negative and significant relationship with the amount of homework done and with time management. The amount of homework done was positively and significantly related to time management and to the amount of time spent on homework, although in the latter case, the relationship was weaker. On another hand, there was no statistically significant relation between the amount of time spent on homework and time management.

**Table 1:** Means, standard deviations, skewness, kurtosis, and correlation matrix of the target variables.

	1	2	3	4	5	6
1. Prior academic achievement	-					
2. Deep approach	0.15^∗∗^	-				
3. Superficial approach	-0.32^∗∗^	-0.17^∗∗^	-			
4. Amount HW	0.33^∗∗^	0.34	-0.16^∗∗^	-		
5. HW time spent	-0.01	-0.01	-0.00	0.10^∗^	-	
6. HW time management	0.25^∗∗^	0.45^∗∗^	-0.22^∗∗^	0.38^∗∗^	-0.02	-
*M*	3.14	4.01	2.62	4.63	2.52	4.06
*SD*	1.18	0.81	0.93	0.73	1.15	0.94
Skewness	-0.25	-0.88	0.48	-2.29	0.59	-1.17
Kurtosis	-0.89	0.59	-0.25	5.21	-0.35	1.52


*^∗^p < 0.05. ^∗∗^p < 0.01.*

### Differences in Motivational and Cognitive Engagement Depending on Prior Academic Achievement (Controlling for the Effect of Grade and Gender)

After controlling for the effects of grade [λ_Wilks_ = 0.926, *F*(2,510) = 20.39, *p* < 0.001, $\eta_{p}^{2}$ = 0.074] and gender [λ_Wilks_ = 0.997, *F*(2,510) = 0.72, *p* = 0.489, $\eta_{p}^{2}$= 0.003], the results revealed statistically significant differences in the set of variables related to motivational and cognitive engagement as a function of the different levels of prior academic achievement [λ_Wilks_ = 0.899, *F*(2,510) = 13.97, *p* < 0.001, $\eta_{p}^{2}$= 0.052]. The effect size was medium.

Taking into account the data on each dependent variable considered individually, there were statistically significant differences depending on the level of students’ prior academic achievement in the shallow approach [*F*(2,511) = 20.95, *p* < 0.001, $\eta_{p}^{2}$= 0.095] and the deep approach [*F*(2,511) = 4.01, *p* < 0.05, $\eta_{p}^{2}$= 0.0015]. In the former case, the effect size was medium and, in the latter case, it was small. In addition, as can be seen, only the grade covariate was significant, with a medium effect size.

**FIGURE 1:**
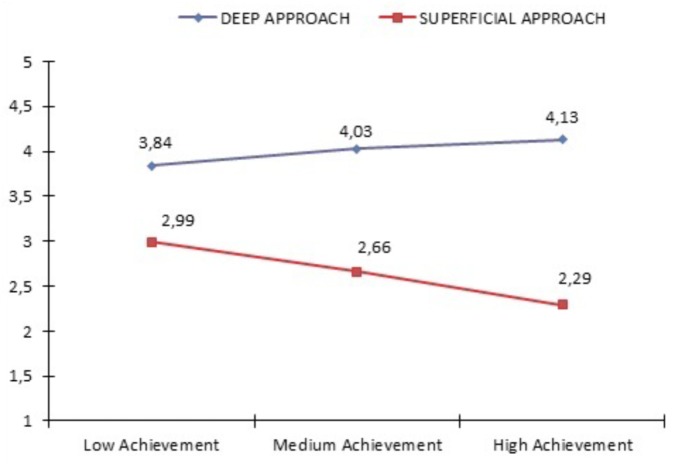
Graphic representation of the mean values in motivational and cognitive engagement (deep approach, shallow approach) as a function of the levels of prior academic achievement.

As can be observed in Table 2 and Figure 1, as the levels of achievement rose, there was a decrease in the use of a shallow approach and an increase in the use of a deep approach.

**Table 2:** Descriptive statistics (mean, standard deviation) corresponding to each of the levels of prior academic achievement in the variables related to motivational and cognitive engagement (approach to homework).

Academic Achievement								
	Low		Medium		High		Total	

	*M*	*DT*	*M*	*DT*	*M*	*DT*	*M*	*DT*
Deep approach	3.84	0.91	4.03	0.74	4.13	0.70	4.01	0.81
Superficial approach	2.99	0.95	2.66	0.94	2.29	0.76	2.62	0.93


### Differences in Behavioral Engagement as a Function of Prior Academic Achievement (Controlling for the Effect of Grade and Gender)

After controlling for the effects of grade [λ_Wilks_ = 0.937, *F*(3,509) = 11.33, *p* < 0.001, $\eta_{p}^{2}$= 0.063] and gender [λ_Wilks_ = 0.993, *F*(3,509) = 1.14, *p* = 0.331, $\eta_{p}^{2}$= 0.007], the results showed statistically significant differences in the set of variables related to behavioral engagement as a function of the different levels of prior academic achievement [λ_Wilks_ = 0.888, *F*(3,509) = 10.42, *p* < 0.001, $\eta_{p}^{2}$= 0.058]. The effect size was medium. Also in this case, only the grade covariate was significant, with a medium effect size.

Taking the data on each dependent variable considered individually, as a function of the level of students’ prior academic achievement, there were statistically significant differences in amount of homework done [*F*(2,511) = 27.51, *p* < 0.001, $\eta_{p}^{2}$ = 0.097] and the optimization of the time spent on homework [*F*(2,511) = 13.28, *p* < 0.05, $\eta_{p}^{2}$= 0.049]. In both cases, the effect size was medium, although the former was quite high. On another hand, there were no statistically significant differences in the time spent on homework [*F*(2,511) = 0.39, *p* = 0.678; $\eta_{p}^{2}$= 0.002] as a function of prior achievement.

**FIGURE 2:**
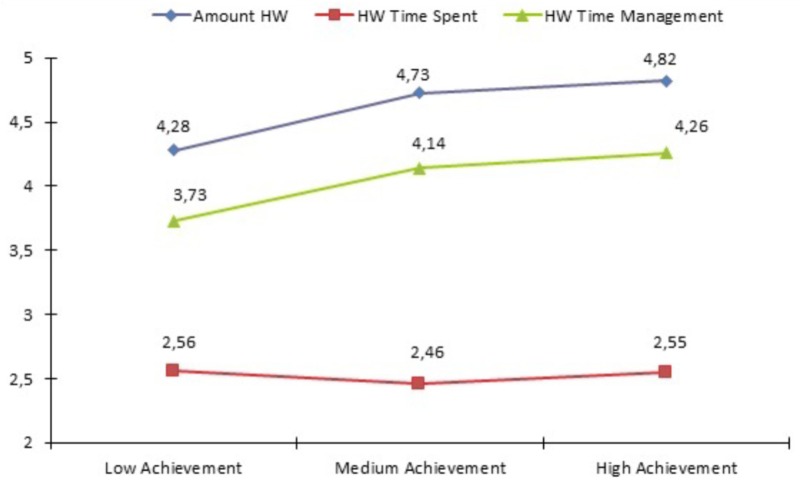
Graphic representation of the mean values of the variables associated with behavioral engagement (amount of homework done-Amount HW-, time devoted to homework-HW Time Spent- and optimization of the time spent on homework-HW Time Management) as a function of the levels of prior academic achievement.

As can be seen in Table 3 and Figure 2, the results indicated that, as prior academic achievement levels rose, there was a progressive increase in the amount of homework done and the optimization of the time devoted to homework. On another hand, there were no statistically significant differences as a function of prior achievement in the time spent on homework.

**Table 3:** Descriptive statistics (mean, standard deviation) corresponding to each of the levels of prior academic achievement in the variables related to behavioral engagement.

	Academic Achievement							
	
	Low		Medium		High		Total	
				
	*M*	*DT*	*M*	*DT*	*M*	*DT*	*M*	*DT*
Amount HW	4.28	0.97	4.73	0.55	4.82	0.50	4.62	0.73
HW Time spent	2.56	1.20	2.46	1.04	2.55	1.20	2.52	1.15
HW Time management	3.73	1.12	4.14	0.84	4.26	0.77	4.06	0.94


## Discussion

The results of this study highlight the relationships between prior academic achievement and students’ degree of cognitive and motivational homework engagement. In fact, higher academic achievement levels are associated with a greater increase in the use of a deep approach and a decrease in a shallow approach to homework.

In addition, prior achievement is also linked with students’ behavioral homework engagement. Thus, higher levels of academic achievement are associated with a greater amount of teacher-assigned homework done and a better optimization of the time spent on homework. On another hand, different levels of prior achievement do not appear to be associated with differences in the amount of time students spend doing homework.

These results indicate that prior experiences of academic success have positive consequences on students’ degree of engagement with homework. These success experiences are a powerful source at the motivational, cognitive and behavioral level, as they not only generate students’ greater confidence in their own abilities, but are also a real antidote for low self-efficacy beliefs and low motivation toward learning. In contrast, prior experiences of failure decrease students’ confidence in their abilities, demotivating them to learn and leading them to avoid engaging in study activities (Bandura, 1993).

In this line, the findings of this study suggest that past successful experiences contribute to generate deeper and less shallow modes or approaches to homework. This finding leads to some educational implications of consideration, taking into account that the deep focus on homework is associated with a high desire to learn and understand the content of the tasks, and to relate the content with students’ prior knowledge (Doménech and Gómez, 2011; Valle et al., 2015b), and, usually, it represents high academic achievement (Bembenutty and White, 2013; Núñez et al., 2014). However, students who use a shallow approach conceive homework as a necessary imposition to achieve other goals. Therefore, their main objective is to complete it as soon as possible, and their greatest concern is to bring it to class completed, in order to avoid reproaches or to please the teacher’s demands, but not with the priority aim of learning (Valle et al., 2015b). As a result, they tend to obtain low academic results (Rosário et al., 2010).

Therefore, the link established between high prior academic achievement, increase of a deep approach to homework and decrease of a shallow approach is one of the keys to achieving an optimum and high quality homework performance, where the students’ degree of cognitive and motivational engagement meets the necessary requirements to ensure that homework is a useful and beneficial tool to strengthen and improve the learning processes and consequently, students’ results. In fact, a deep learning approach is associated with higher quality results (Cano et al., 2014). And also in the case of homework, the approach employed conditions not only the process of doing homework and the results, but also students’ level of homework engagement.

Prior academic achievement is also related to the degree of behavioral homework engagement. Specifically, higher levels of prior achievement are associated with a greater amount of homework done and a better optimization of the time spent on homework. The positive relationship between the amount of homework done and academic achievement has been found in several research studies on homework (Cooper, 1989; Cooper et al., 1998; Trautwein et al., 2002; Valle et al., 2015a).

Within behavioral homework engagement, another variable is the time spent on homework. In this case, a difference has been established between the amount of time spent on homework and the quality of that time (time optimization) The results of this study indicate that there is only a positive and statistically significant relationship between prior achievement and the optimization of the time spent on homework, but there is no relationship with the amount of time spent on homework. These results are in line with other prior works (see, e.g., Trautwein et al., 2006; Flunger et al., 2015) in which it was found that students who spend more time on homework are not necessarily better students, but rather may be students who have greater difficulties, concentration problems, or who are not sufficiently motivated. The effort that a student makes doing homework is not necessarily related to the amount of time that he or she takes to do it (Trautwein et al., 2015. However, our results emphasize the relevance of prior academic achievement in effective time management when doing homework. This finding is consistent with other studies (e.g., Núñez et al., 2015a) in which it was found that optimization of the time devoted to homework was the variable that best predicted students’ academic achievement.

In this way, it can be deduced that the study of the relationship between prior achievement and time devoted to homework should take into account other matters related to the process of homework and should contemplate other variables—perhaps more relevant to this process—such as, for example, all those related to time management skills. In fact, the relationship between the amount of homework done, the time devoted to it, and academic achievement may be moderated by the actual optimization of the time students spend on homework (Valle et al., 2017). In the same vein, it should be noted that students who manage their homework time well are the ones who engage in it more deeply; hence, homework time optimization is more decisive than the amount of time devoted to homework (Valle et al., 2015b). Additionally, the criteria that we have used to determine the three levels of prior achievement should be taken into account. Although percentiles are a common grouping criterion in educational research, they can limit the statistical power of the results obtained.

Despite this limitations, the results of this work allow us to establish a clear relationship between prior academic achievement and students’ degree of motivational, cognitive, and behavioral homework engagement. Previous experiences of academic success are associated with certain indicators that reveal the quality of the process of doing homework. These indicators are related to greater use of a deep approach to homework, with a better and more efficient management of the time devoted to homework and also with a greater amount of teacher-assigned homework done. Probably, these quality indicators of the process of doing homework will also have positive effects on the students’ overall academic achievement and their degree of engagement in their learning process.

Future research should clarify some of the results that seem to call into question, for example, the relationship between the time spent on homework and achievement. In fact, although some multilevel studies show that, at the individual level, the time spent on homework has little incidence on academic achievement, when measured at the classroom level, the results are positive (Fernández-Alonso et al., 2014). The possible effect on homework of the variable “grade” should also be reviewed in greater detail, especially through longitudinal studies contemplating the possible change in the different variables involved in homework as students go on to higher grades. In this sense, future works should take into account, not only the three grades contemplated in this study, but also the first three years of primary education. Finally, socioeconomic status was not measured in our study. Consequently, future works should analyze the specific role played by students’ socioeconomic status in the relationship between prior achievement and homework engagement.

## Author Contributions

IP, IE, and CF contributed to conception and design of the study and wrote the first draft of the manuscript. AS organized the database. IE and CF performed the statistical analysis. AdC, AS, and MG-S wrote sections of the manuscript. All authors contributed to manuscript revision, read, and approved the submitted version.

## Conflict of Interest Statement

The authors declare that the research was conducted in the absence of any commercial or financial relationships that could be construed as a potential conflict of interest.
